# Mitochondrial Structure and Function Are Disrupted by Standard Isolation Methods

**DOI:** 10.1371/journal.pone.0018317

**Published:** 2011-03-28

**Authors:** Martin Picard, Tanja Taivassalo, Darmyn Ritchie, Kathryn J. Wright, Melissa M. Thomas, Caroline Romestaing, Russell T. Hepple

**Affiliations:** 1 Department of Kinesiology, McGill University, Montreal, Quebec, Canada; 2 Muscle and Aging Laboratory, Faculty of Kinesiology and Faculty of Medicine, University of Calgary, Calgary, Alberta, Canada; 3 Laboratoire de Physiologie Intégrative, Cellulaire et Moléculaire, Université de Lyon, Villeurbanne, France; Texas A&M University, United States of America

## Abstract

Mitochondria regulate critical components of cellular function via ATP production, reactive oxygen species production, Ca^2+^ handling and apoptotic signaling. Two classical methods exist to study mitochondrial function of skeletal muscles: isolated mitochondria and permeabilized myofibers. Whereas mitochondrial isolation removes a portion of the mitochondria from their cellular environment, myofiber permeabilization preserves mitochondrial morphology and functional interactions with other intracellular components. Despite this, isolated mitochondria remain the most commonly used method to infer *in vivo* mitochondrial function. In this study, we directly compared measures of several key aspects of mitochondrial function in both isolated mitochondria and permeabilized myofibers of rat gastrocnemius muscle. Here we show that mitochondrial isolation i) induced fragmented organelle morphology; ii) dramatically sensitized the permeability transition pore sensitivity to a Ca^2+^ challenge; iii) differentially altered mitochondrial respiration depending upon the respiratory conditions; and iv) dramatically increased H_2_O_2_ production. These alterations are qualitatively similar to the changes in mitochondrial structure and function observed *in vivo* after cellular stress-induced mitochondrial fragmentation, but are generally of much greater magnitude. Furthermore, mitochondrial isolation markedly altered electron transport chain protein stoichiometry. Collectively, our results demonstrate that isolated mitochondria possess functional characteristics that differ fundamentally from those of intact mitochondria in permeabilized myofibers. Our work and that of others underscores the importance of studying mitochondrial function in tissue preparations where mitochondrial structure is preserved and all mitochondria are represented.

## Introduction

Mitochondria are key regulators of cellular function and hence their dysfunction is implicated in the pathogenesis of many diseases [1], [2], [3], [4] and the very process of aging itself [5], [6]. For this reason, the study of mitochondrial function has become central to a wide variety of clinical and basic science research. A powerful tool to investigate mitochondrial function was developed more than fifty years ago by Chance and Williams (1956), involving the isolation of mitochondria from skeletal muscle. This method allows the recovery of a relatively pure mitochondrial fraction, through first homogenizing a fresh muscle sample and then purifying the mitochondria through a series of differential centrifugation steps [7]. Notably, this *in vitro* approach allowed elucidation of the nature of the tricarboxilic cycle (Krebs cycle) in the 1960's [8] and it continues to this day to be used widely to study a variety of aspects of mitochondrial biology in skeletal muscle, including mitochondrial permeability transition pore (mPTP) function [9], [10], respiratory capacity [9], [10], [11], reactive oxygen species (ROS) production [10], [12], [13], mitochondrial protein import and assembly [14], [15] and the mitochondrial genome and proteome [16]. Despite the widespread adoption of this technique, standard isolation methods retrieve a low (generally 20–40% of total) fraction of the total mitochondrial content from muscle [17], [18], [19], [20]. For this reason, isolated mitochondria studies necessitate relatively large amounts of fresh tissue and have been suggested to lead to potential bias because of selective representation of the entire mitochondrial pool [21].

Another experimental method to study mitochondrial function in muscle was subsequently developed by Saks and colleagues (1998), which involved the preparation of permeabilized myofibers, or skinned fibers. This method entails manual separation of muscle myofibers, followed by selective permeabilization of the sarcolemma, leaving ≥95% of all mitochondria intact within the normal cytoarchitechtural environment [22], [23], [24]. Although this method is gaining in popularity, there remains limited data comparing this approach to isolated mitochondria, with studies thus far having focused only on respiration [18], [21], [23].

**Figure 1:**
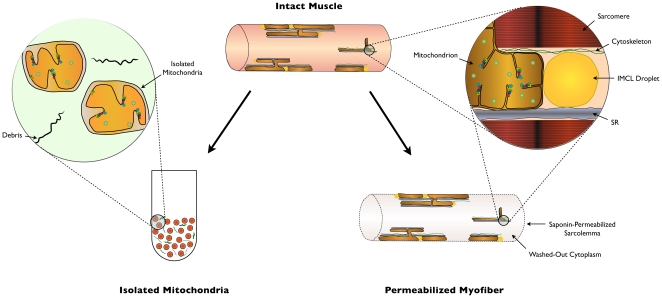
Simplified schematic representation of the presumed structural effect of preparing isolated mitochondria and permeabilized myofibers from skeletal muscle. Mitochondria exhibit a three-dimensional reticular morphology in intact myofibers and physically interact with surrounding mitochondria and other structures (e.g., sarcoplasmic reticulum (SR), cytoskeleton, lipid droplets). In isolated mitochondria, only a fraction of total mitochondria are recovered, which are morphologically distinct from that of intact muscle and lack functional interactions with surrounding cellular structures. In permeabilized myofibers, the sarcolemma is selectively and partially dissolved and the cytoplasm is washed out (for specific depiction of membrane permeabilization process, see [22]). This method provides access through diffusion to mitochondria located within the cells, where the intracellular cytoarchitectural environment is preserved and >95% of mitochondria are present.

In addition to concerns over selective and low yield of mitochondria via standard isolation procedures, other concerns relate to the disruption of mitochondrial three-dimensional network or reticular structure [25], [26] and lack of interaction with other cellular compartments (e.g., sarcoplasmic reticulum, cytoskeleton, lipid droplets) following the isolation of mitochondria [21], [23]. Recent evidence also suggests that mitochondrial morphology is closely associated to various functional aspects [27], [28]. As such, it seems reasonable to postulate that standard mitochondrial isolation procedures, which by definition must disrupt mitochondrial structure due to the mechanical nature of the homogenization and centrifugation procedures (Figure 1), could have quite dramatic effects on mitochondrial function.

In this study, we took advantage of the preservation of mitochondrial structure in permeabilized myofibers [21], [23], [24] to determine the impact of isolation on normal mitochondrial function. Specifically, using these two methods in parallel, we systematically measured three key indices of mitochondrial function: sensitivity of the mPTP opening to a Ca^2+^ challenge, respiration, and H_2_O_2_ generation (a surrogate measure of ROS production) in samples prepared from the same muscles of rats. To provide insights into any functional differences observed, we also examined mitochondrial structure and electron transport chain stoichiometry.

## Results

### Isolated mitochondrial morphology and sensitivity to mPTP opening

**Figure 2:**
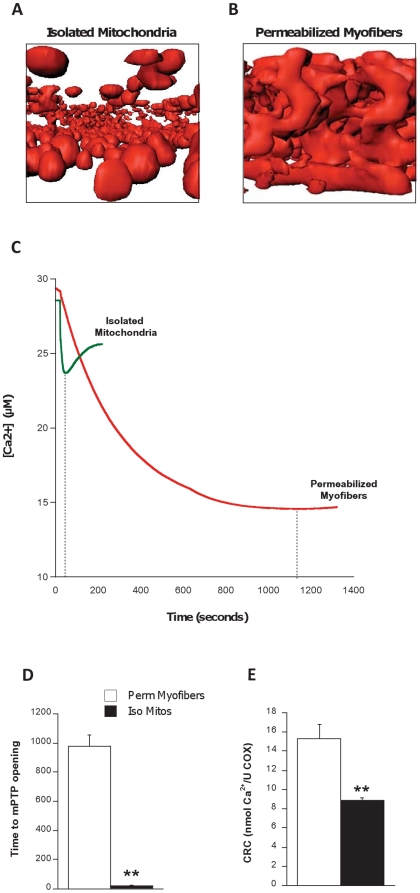
Altered mitochondrial morphology and increased mPTP sensitivity to Ca^2+^ in isolated mitochondria. (**A**) Three-dimensional reconstruction of isolated mitochondria and (**B**) permeabilized myofiber incubated with MitoTracker Red and imaged using confocal microscopy (see also Video S1 and Video S2). (**C**) Representative traces of calcium uptake by mitochondria within permeabilized myofibers and isolated mitochondria. Vertical dotted lines indicate time taken as mPTP opening. (**D**) Quantification of time to mPTP opening in mitochondria from both types of preparations. (**E**) Quantification of calcium retention capacity (CRC) calculated as the amount of Ca^2+^ taken by mitochondria before opening of the mPTP. N = 8 animals per group, values are means ± s.e.m. ** = p<0.01.

We first examined by confocal microscopy the morphology of isolated mitochondria from the mixed region of gastrocnemius muscle using Mitotracker Red. Contrary to well-documented reticular mitochondrial morphology in skeletal muscles [25], [26], [29], [30], isolated organelles showed a consistent spherical appearance and a relatively homogenous size distribution (Figure 2A, see also Video S1 and Figure S1) compared to that observed in permeabilized myofibers (Figure 2B, see also Video S2). We also measured mitochondrial Ca^2+^ uptake, with strikingly different dynamics observed between preparations (Figure 2C). The Ca^2+^ uptake data indicate two major points. First, upon Ca^2+^ stress, most or all isolated mitochondria undergo mPTP opening almost simultaneously (within 5–10 seconds), whereas in permeabilized myofibers, mitochondria exhibiting a broad range of sensitivities undergo mPTP opening at different times (several minutes apart), causing a gradual and progressive inversion of the Ca^2+^ uptake signal. Second, we also found that time to mPTP opening was 98% shorter in isolated mitochondria compared to permeabilized myofibers (21 vs 977 seconds, respectively) (Figure 2D) and the amount of Ca^2+^ necessary to trigger opening of the mPTP was 42% lower in the isolated preparations (Figure 2E), demonstrating a marked sensitization of the mPTP to a Ca^2+^ challenge in isolated mitochondria. To permit adequate comparison between both preparations, all functional data was normalized per enzymatic activity of cytochrome c oxidase (COX) measured *a posteriori* in mitochondrial isolates and homogenates of permeabilized myofibers from each animal.

### Mitochondrial respiration

**Figure 3:**
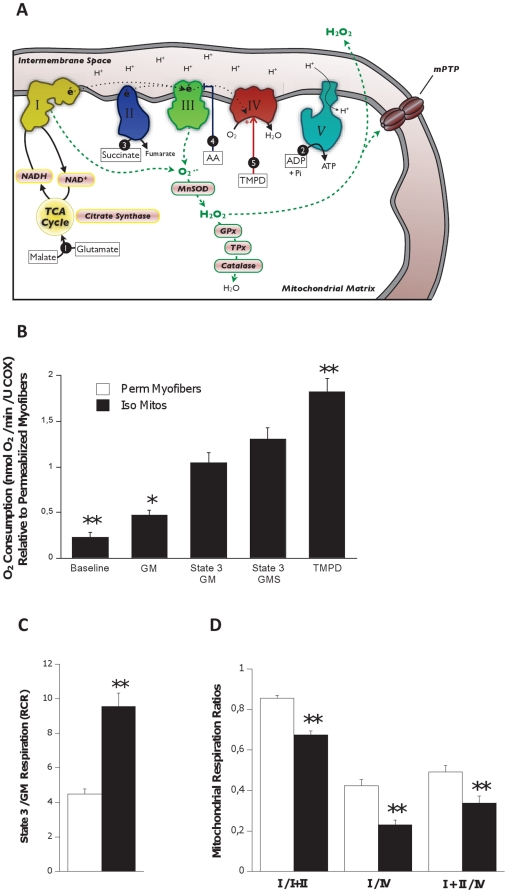
Quantitative and qualitative alterations of mitochondrial respiration in isolated mitochondria. (**A**) Schematic diagram of the relevant mitochondrial components involved in mitochondrial respiration and antioxidant defenses. 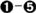 represent the sequence and site of action of each substrate added to the respirometry assay. ***Bold italicized*** items are matrix components which may be partially lost during mitochondrial isolation. (**B**) Mitochondrial oxygen consumption with sequential substrate addition protocol in both preparations, expressed relative to permeabilized myofibers. Baseline – permeabilized myofibers or isolated mitochondria without substrate; GM – Glutamate-Malate; State 3 GM – GM + ADP; State 3 GMS – State 3 GM + Succinate; TMPD – State 3 GMS + Antimycin A (AA) + TMPD + Ascorbate. (**C**) Respiratory control ratio (RCR) for both preparations. (**D**) Mitochondrial respiration ratios calculated for both preparations, representing the relative activity of complexes I, II and IV. *Abbreviations*: I, II, III, IV, IV – electron transport chain complexes I to IV, and ATP synthase (V); NAD^+^ – nicotinamide adenine nucleotide; NADH – reduced NAD^+^; TCA – tricarboxilic acid cycle; MnSOD – manganese superoxide dismutase; GPx – glutathione peroxidase; TPx – thioredoxin peroxidase. N = 8 animals per group, values are means ± s.e.m. * = p<0.05 ** = p<0.01.

To address the effect of experimental preparation on mitochondrial respiration, mitochondrial O_2_ consumption was measured during a sequential substrate addition protocol (schematically illustrated in Figure 3A) and normalized for a marker of mitochondrial content (complex IV activity). Compared to permeabilized myofibers, respiration of isolated mitochondria was lower under basal (77% lower) and state II (GM; 53% lower) conditions (Figure 3B). However, comparison of state 3 respiration, after activation of both complex I (by glutamate and malate) and complex II (by succinate), revealed similar maximal respiration rates between the two methods. Conversely, direct stimulation of Complex IV yielded an 82% higher respiration rate in isolated mitochondria (Figure 3B). Respiratory control ratio (RCR), defined as ratio of state 3 (with ADP) to state 2 respiration (without ADP), was also 1.1-fold higher in isolated mitochondria than in permeabilized myofibers (Figure 3C). To determine the relative activity of each complex, we calculated the stoichiometry of respiration rates between complexes I, II and IV. We found a reduced activity of complex I relative to that of complex II and complex IV in isolated mitochondria compared to permeabilized myofibers (Figure 3D).

### Electron transport chain protein stoichiometry

**Figure 4:**
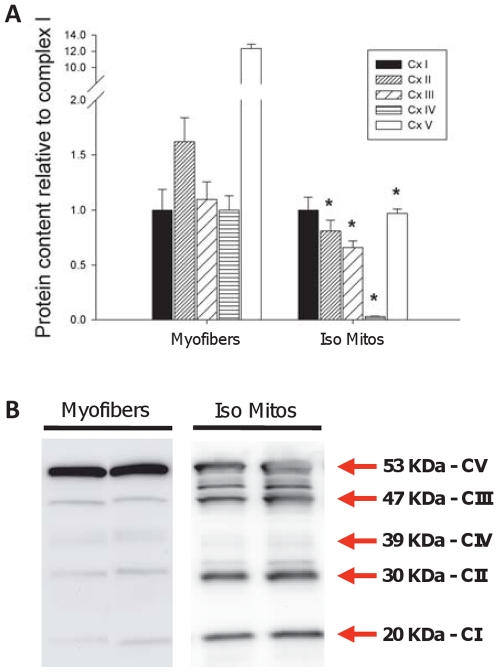
Altered stoichiometry of electron transport chain and ATP synthase complexes in isolated mitochondria. (**A**) Quantification of Western blots probed for the relative abundance of representative subunits of each of the four mitochondrial electron transport chain complexes (I–IV) and the ATP synthase (V). Mean optical density values are expressed relative to complex I within a given preparation. (**B**) Representative Western blots from whole muscle and purified isolated mitochondria. N = 8 animals per group, values are means ± s.e.m. * = p<0.05 ** = p<0.01.

To investigate the relative abundance of electron transport chain complexes between preparations, we performed Western blot experiments. Quantification of blots indicated marked differences in the relative abundance of complex I, II, III and IV between preparations (Figure 4A). Further analysis of the Western blots revealed intermediate protein size bands in isolated mitochondrial preparations, contrasting with the discrete bands corresponding to the predicted molecular weights of the subunits detected by the antibody cocktail in whole muscle (Figure 4B).

### Mitochondrial H_2_O_2_ release

**Figure 5:**
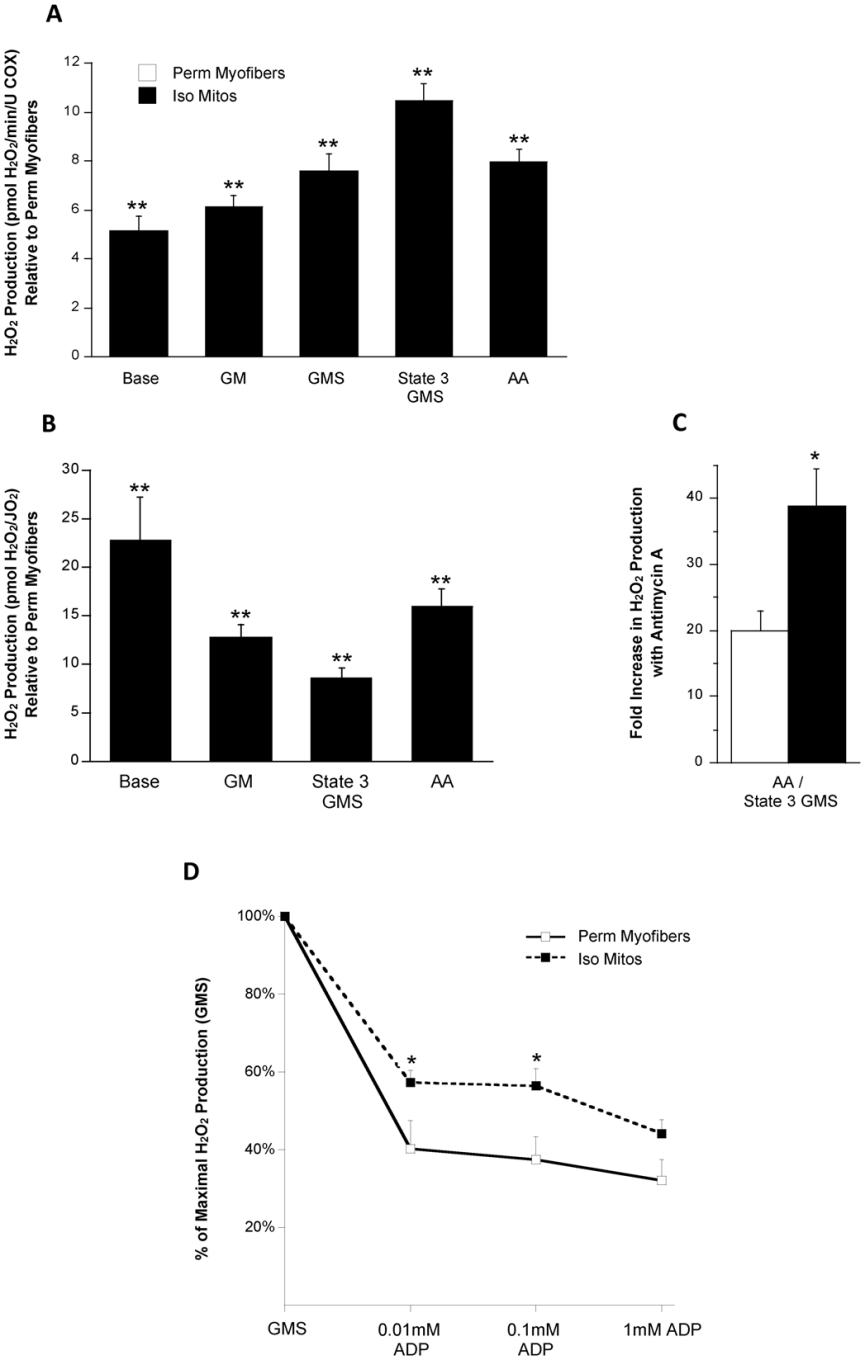
Increased mitochondrial reactive oxygen species production in isolated mitochondria. (**A**) H_2_O_2_ release by mitochondria of both preparations during different activation states, normalized to mitochondrial content (COX activity) and expressed relative to permeabilized myofibers values. (**B**) H_2_O_2_ release by mitochondria, normalized to oxygen consumption and expressed relative to permeabilized myofibers values. (**C**) Effect of adding antimycin A (AA) on maximal H_2_O_2_ production per O_2_ flux (JO_2_). (**D**) Effect of adding increasing amount of ADP on H_2_O_2_ production. N = 8 animals per group, values are means ± s.e.m. * = p<0.05 ** = p<0.01.

To address the effect of mitochondrial isolation on mitochondrial H_2_O_2_ production under different energetic states, we again used a sequential substrate addition protocol. When expressed in relation to a marker of mitochondrial content (complex IV activity), H_2_O_2_ production was 5–10 fold higher in isolated mitochondria than in permeabilized myofibers (Figure 5A) and when expressed per respiration rate and therefore normalized per electron flow within the electron transport chain, H_2_O_2_ production was a staggering 9–23 fold higher in isolated mitochondria, depending upon the substrate conditions (Figure 5B). Furthermore, blockade of electron flow at complex III resulted in a 2-fold greater increase in H_2_O_2_ production in isolated organelles than in permeabilized myofibers (Figure 5C), suggesting a greater propensity of isolated mitochondria to generate ROS under blocked electron flow.

We also investigated the effect of incrementally activating respiration by stepwise addition of increasing concentrations of ADP, allowing H^+^ flux through Complex V and thereby harnessing the membrane potential for ATP production and reducing H_2_O_2_ production. Our results show that ADP addition had a significantly smaller effect in reducing H_2_O_2_ production in isolated mitochondria than in permeabilized myofibers (Figure 5D) providing further evidence of an increased propensity for ROS production by isolated mitochondria.

## Discussion

**Figure 6:**
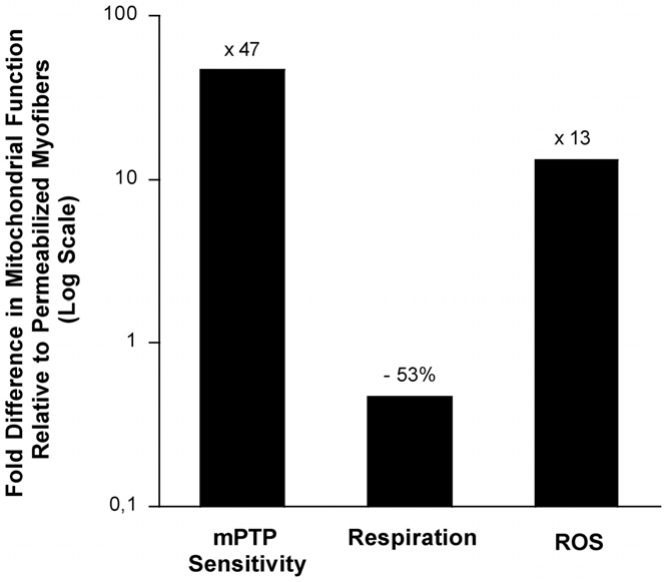
Summary of the functional impact of mitochondrial isolation. Average values in isolated mitochondria are expressed relative to values in permeabilized myofibers. Shown are values under state 2 respiration with glutamate-malate (GM) and normalized for mitochondrial content estimated with COX activity. Relative to permeabilized myofibers, isolated mitochondria exhibit marked sensitivity to mPTP opening, expressed as the inverse of time required for mPTP opening upon Ca^2+^ challenge. Oxygen consumption under state 2 respiration normalized for mitochondrial content is significantly lower in isolated mitochondria than in permeabilized myofibers. Finally, reactive oxygen species production (measured as H2O2 release) normalized to O2 flux is higher than in permeabilized myofibers.

The study of skeletal muscle mitochondrial function has historically involved two primary methodologies: isolated mitochondria, or permeabilized myofibers. Although prior studies have provided important initial evidence of significant alterations of respiratory function by mitochondrial isolation compared to the intact mitochondria in permeabilized myofibers [18], [21], [23], neither the characterization across a wide spectrum of respiratory states, nor other important aspects of mitochondrial function, such as mPTP function and H_2_O_2_ production, have yet been reported. This prompted us to compare different indices of routinely measured aspects of mitochondrial function of mitochondria prepared with these two methods. Strikingly, our data reveal for the first time that following mechanical homogenization, isolated mitochondria exhibit spherical homogenous morphology, increased sensitivity to Ca^2+^-induced mPTP opening, altered respiratory capacity, altered electron transport chain protein stoichiometry, and dramatically higher levels of H_2_O_2_ release compared to mitochondria from permeabilized myofibers (Figure 6). As such, our results provide novel insights into the marked magnitude of alterations induced by mitochondrial isolation methods in every aspect we examined (structure, function, electron transport chain stoichiometry). On this basis, we suggest that isolated mitochondria may better represent stressed organelles than mitochondria functioning under normal circumstances *in vivo*.

### Role of Fragmented Morphology in Isolated Mitochondrial Function

At the point in time when mitochondrial isolation methods were first developed, the notion of mitochondrial structure in skeletal muscle was that of a roughly spheroid organelle, based mainly upon their endosymbiotic origin as autonomous bacteria [28] and the appearance of these organelles in two dimensional electron micrograph images. However, three dimensional scanning electron microscopy studies of skeletal muscle mitochondria have since revealed that their structure is diverse and characterized by an irregular shaped tubular network of varied size and complexity [25], [26], [29], [30]. Furthermore, mitochondrial structure is dynamically regulated by processes of fusion and fission [2], [27], [31], and these changes in structure induce alterations in mitochondrial apoptotic signaling, respiration and ROS production that are relevant and specific to metabolic conditions within the cell [27], [32].

On the basis of the above points, it is logical to expect that standard mitochondrial isolation methods should disrupt mitochondrial structure from its *in vivo* state, and depending upon the degree of that disruption, induce corresponding changes in many aspects of mitochondrial function. Consistent with this idea, here we show that in contrast to the heterogenous mitochondrial structure of skeletal muscle evident *in vivo*, the three-dimensional morphology of isolated mitochondria was relatively homogenous and consisted only of spherical organelles (Videos S1 and S2), showing that significant fragmentation of mitochondrial structure occurs upon isolation. Interestingly, mitochondrial fission-induced fragmentation is a critical event facilitating the release of pro-apoptotic factors and mPTP opening [33], [34], [35]. Thus, our observation that isolated mitochondria exhibited markedly enhanced sensitivity to mPTP opening could be a direct consequence of the fragmented mitochondrial morphology we observed, although it is important to note that we have no basis for comparing the degree of fragmentation in the isolates we studied to that seen in mitochondria undergoing fission *in vivo*.

In our experiments, most isolated mitochondria underwent mPTP opening quasi-simultaneously, which contrasts not only with our results in permeabilized myofibers, but also with evidence from live cell experiments where time to mPTP opening is markedly heterogenous [36]. Thus, both the calcium retention capacity and *timing* of mPTP opening were altered in isolated mitochondria. Furthermore, because mitochondrial fission has been shown to be an upstream causal factor of elevated ROS production in cells challenged with hyperglycemic conditions [37], [38], the severe degree of mitochondrial fragmentation evident in isolates could contribute to the dramatic increase in H_2_O_2_ production that we observed. Since ROS is a known sensitizing stimulus for mPTP opening *in vivo* [39], this increase in H_2_O_2_ production may also contribute to the marked sensitization of mPTP opening to Ca^2+^ in the isolated mitochondria.

We note that both the changes we observed in mPTP function (47-fold) and H_2_O_2_ generation (13-fold) appear substantially greater than those occurring *in vivo* following mitochondrial fission. For example, increases in mPTP sensitivity induced by overexpression of the pro-fission protein hFis1 [35] or increases in ROS production induced by hyperglycemia-induced mitochondrial fission [37], [38] are in the range of 50 to 300% above baseline. This difference in magnitude suggests that the functional outcomes of mitochondrial isolation are quantitatively more severe than the regulated fission processes. This may be a reflection of differences in the degree of fragmentation (perhaps more severe disruption is evident in the isolates than fissioned mitochondria *in vivo*), or other factors related to the process by which the mitochondria are mechanically homogenized, as discussed in the next paragraph. Importantly, we do not believe that the striking magnitude of the differences we observed relates to experimental problems with either method. In making this statement, we note that the RCR is typically regarded as a good indicator of mitochondrial preparation quality [22], [40] and RCR has been consistently reported to be about two-fold higher in isolated mitochondria (e.g., Chabi *et al.*, 2008) than permeabilized myofibers (about 10 vs 5, respectively) [21], [22]. Accordingly, we found RCR values consistent with these standards, indicating that both of our methods yielded adequately coupled and high quality preparations with the expected differences in RCR. Similarly, the values we observed for state III respiration in both preparations are in the upper range of values reported in the literature [10], [21], [41]. Therefore, we have no basis for believing that the marked differences we have observed between preparations relates to problems in successfully applying these methods.

### Factors Unrelated to Morphology in Isolated Mitochondrial Function

In addition to the fragmentation of mitochondrial morphology noted above, there are many other factors which could contribute to the profound alteration of key aspects of mitochondrial function following their isolation. Amongst these, selective loss of soluble mitochondrial matrix constituents or dilution of matrix content due to a transient rupture/resealing of mitochondrial membranes during isolation has previously been suggested to occur following mitochondrial isolation [42]. Disruption of the binding between mitochondrial proteins and surrounding cytoskeletal elements has also been demonstrated to account for subtle differences in the affinity for ADP during mitochondrial respiration [23]. In addition to these factors, we suggest that during transient rupture/reseal of mitochondrial membranes, consequent to fragmentation of the irregular mitochondrial tubular network into spheroid particles (Figure 1), the protease Nagarse used in the isolation medium to maximize the recovery of intermyofibrillar mitochondria enters the mitochondria where it exerts insidious and non-specific proteolytic activity. These two possibilities are supported by two findings. First, we observed a preferential deficiency of respiration with Complex I substrates, where there is dependence upon substrate oxidation by Krebs cycle enzymes (matrix located) and electron transfer by the coenzyme nicotinamide adenine nucleotide (NAD^+^; also located in the matrix) (see Figure 2A), compared with the direct oxidation of succinate by succinate dehydrogenase (complex II) and direct stimulation of cytochrome c oxidase (complex IV) by TMPD, which operate via more direct mechanisms that are not dependent upon matrix constituents. Second, we detected the presence of multiple intermediate size protein bands on Western blots of isolated mitochondria but not permeabilized myofibers, indicating partial cleavage of a fraction of the probed proteins into smaller fragments by the protease Nagarse. This finding is consistent with previous reports that Nagarse remains unexplainably “associated” with mitochondria isolated from brain [43] and skeletal muscle [44], even after several cycles of wash. Thus, preferential deficiency of complex I-driven respiration and higher rates of H_2_O_2_ release by the mitochondria could result from the loss of endogenous oxidative (krebs cycle, ETC complex subunits) and antioxidant enzymes (see Figure 2A), respectively. This may occur by diffusion of soluble matrix proteins outside of mitochondria or by subsequent proteolytic activity of Nagarse in the matrix space. However, these possibilities need to be directly tested in subsequent studies.

### Summary

In summary, based on our data, we suggest that the functional alterations induced by mitochondrial isolation that we observed here are likely attributable to a combination of three factors: i) severely fragmented mitochondrial morphology due to the disruption of the irregular tubular network induced by mechanical homogenization; ii) loss of soluble proteins and of other molecules from the mitochondrial matrix; and iii) degradation of mitochondrial matrix proteins by the insidious action of Nagarse present in the isolation buffer. Future studies will be necessary to test these and other possible mechanisms. Although on the one hand our results warrant caution in the interpretation of data collected using isolated organelles, they also suggest that quantifying the impact of mitochondrial isolation on mitochondrial function could be used to interrogate the stress resistance of the mitochondrion under a variety of pathophysiological situations. This, and other novel applications of mitochondrial isolation methods, should be considered in future studies.

## Methods

### Ethics Statement

All procedures were conducted with approval from the University of Calgary Animal Care Committee, protocol ID BI09R-11.

### Surgical Methods

8 month old male Fischer 344 x Brown Norway F1-hybrid (F344BN) rats were obtained from the colony maintained by the National Institute on Aging. Rats were anesthetized with 55–65 mg x kg^−1^ sodium Pentobarbital intraperitoneal (i.p.). The left and right Gastrocnemius (Gas) muscles from 8 animals were carefully dissected and placed into ice-cold stabilizing Buffer A (in mM: 2.77 CaK_2_EGTA, 7.23 K_2_EGTA, 6.56 MgCl_2_, 0.5 Dithiothreitol [DTT], 50 K-MES, 20 imidazol, 20 taurine, 5.3 Na_2_ATP, 15 phosphocreatine, pH 7.3 at 4°C). To facilitate greater homogeneity between samples obtained from a given Gas muscle, the highly oxidative red region and highly glycolytic white region of this muscle were removed to leave the mixed region of this muscle. The mixed Gas was then divided equally for mitochondrial isolation and permeabilized myofiber preparations.

### Mitochondrial Isolation

Mitochondrial isolation was performed using standard homogenization, protease digestion and differential centrifugation methods, similar to those described in [7]. Mixed Gas muscle was weighed and placed in 20 ml ice-cold mitochondrial extraction buffer (in mM: 100 sucrose, 50 KCL, 5 EDTA, 2 KH_2_PO_4_, 50 Tris-base, pH 7.4 at 4°C) and subsequently minced manually with fine scissors. All steps were performed at 4°C. Minced tissue was homogenized at 600 rpm with a motor driven Teflon Potter Elvehjem pestle (6 up and down pulses), incubated with 1 mg x g^−1^ nagarse protease (Sigma, P8038) for 5 min, diluted further with another 20 ml extraction buffer and homogenized again at 600 rpm (4 up and down pulses). The homogenate was centrifuged at 1000 *g* for 10 min, after which the mitochondria-rich supernatant was filtered through cheesecloth and the pellet discarded. Mitochondria were then pelleted by centrifugation at 8000 *g* for 10 min and gently re-suspended in re-suspension buffer (in mM: 100 sucrose, 50 KCL, 05 EDTA, 2 KH_2_PO_4_, 50 Tris-base, pH 7.4 at 4°C), centrifuged again at 8000 *g* for 10 min, and the final pellet gently re-suspended in 600 µl of re-suspension buffer. Mitochondrial protein concentration was measured spectrophotometrically using the bicinchoninic acid (BCA) assay (Thermo Scientific, 23225). Isolated mitochondria were used fresh for functional measurements. A portion of fresh isolated mitochondria was frozen for Western blots and enzymatic activity measurements.

### Preparation of permeabilized myofibers

Dissection and permeabilization of myofibers with saponin was performed according to methods described by Kuznetsov et al. (2008), and as we have described previously [45]. Briefly, whole muscles were trimmed of connective tissue and manually teased into small myofiber bundles, under a binocular microscope, into a fine mesh where individual threads are 2–5 single muscle fibers in width, to maximize the surface area in contact with the buffers [21]. Once dissection was completed, myofibers were placed in Buffer A supplemented with 0.05 mg x ml^−1^ saponin and incubated at low rocking speed for 30 min to allow selective permeabilization of the sarcolemma. Following incubation, permeabilized myofibers were subjected to three x 10 min rinses in Buffer B (in mM: 2.77 CaK_2_ EGTA, 7.23 K_2_EGTA, 1.38 MgCl_2_, 3.0 K_2_HPO_4_, 0.5 dithiothreitol, 20 imidazole, 100 K-MES, 20 taurine, pH 7.3, at 4°C) supplemented with fatty acid free bovine serum albumin (BSA: 2 mg x ml^−1^). Permeabilized myofibers for respiration experiments were kept in Buffer B on ice until use, where small 4–6 mg (wet weight) packages of permeabilized bundles of myofibers were blotted and pre-weighed prior to respiration experiments. The same procedure was also applied to H_2_O_2_ production and Ca^2+^ retention capacity measurements.

### Imaging of isolated mitochondria and permeabilized myofibers

Freshly isolated mitochondria were diluted to a protein concentration of about 2.5 mg x ml^−1^ (see Figure S1 for specific value) and incubated with 16.7 µM of Mitotracker Red CMXROS (Molecular Probes M7512) for 20 min at 30°C. The same conditions were used to label mitochondria in permeabilized myofibers. Ten µl of labeled mitochondria was placed on a glass slide and mounted with a coverslip to be imaged. Excess liquid was extruded, mitochondria were left to settle for 5 minutes, and images from five independent preparations were acquired using a confocal microscope (Olympus Fluoview FV1000, Olympus fluoview version 2.0c software) with a PlanApo N 60x/1.42 oil immersion objective and 1.6 digital zoom (96x final, 1 pixel  = 0.0827 µm). Alexa Fluor 546 excitation settings were used with pinhole size of 110 µm, z-slices of 0.5 or 0.3 µm, and the following laser settings: HV = 369, Gain = 1, Offset = 21. Imaris 7.0 software was used to analyze z-stacks and produce surface renderings, volume and mean fluorescence intensity measurements. Software settings were: smooth deactivated; diameter of largest sphere of 0.7 µm; threshold for background subtraction of 1030 µm^2^; split touching objects enabled; estimated diameter of 0.444 µm; quality threshold above 260; sphericity threshold above 0.550 (94%+ selection). Raw data of confocal imaging experiments is shown in Figure S1. The three-dimensional landscape of isolated mitochondria and permeabilized myofibers can be compared in Videos S1 and S2, respectively.

### Sensitivity to Ca^2+^-induced mitochondrial permeability transition pore opening

Accumulation of Ca^2+^ in the mitochondrial matrix is one of the most important and obligatory triggers for mitochondrial permeability transition pore (mPTP) opening in skeletal muscle and sensitivity to mPTP opening is therefore commonly assessed in isolated mitochondria by determining mitochondrial Ca^2+^ retention capacity (CRC) in the presence of a Ca^2+^ challenge [46]. We prepared “phantom” myofibers without myosin from eight different animals, as described previously [45] and adapted from [21], to measure mitochondrial Ca^2+^ uptake and detect opening of the mPTP. Briefly, a muscle bundle of 4–6 mg wet weight was added to 600 µl of CRC Buffer containing about 30 µM of Ca^2+^ supplemented with (in mM: 5 glutamate, 2.5 malate, 10 Pi, 0.001 Calcium-green 5N and 0.5 nM oligomycin). For isolated mitochondria, about 0.04 mg of protein isolate was added to 1.5 ml of the same buffer. Mitochondrial Ca^2+^ uptake was immediately followed in a fluorometer by monitoring the decrease in extra-mitochondrial Ca^2+^ concentration using the fluorescent probe Calcium-green 5N (Molecular Probes, Eugene, OR, USA) at excitation/emission wavelengths of 505/535 nm, using the FL-solutions software. CalciumGreen™ fluorescence units were used to compute the amount of Ca^2+^ uptake from addition of samples (beginning when signals starts to fall), to the lowest point of the curve upon signal inversion (indicated by the dotted line). Fluorescence signal was converted to [Ca^2+^] using an exponential standard curve established with increasing amounts of Ca^2+^ added to 600 µl of supplemented CRC buffer. Progressive uptake of Ca^2+^ by mitochondria was monitored until mitochondrial Ca^2+^ release caused by opening of the mPTP was observed as the inversion of signal. CRC, a reliable index of mPTP sensitivity [47], was calculated as total amount of Ca^2+^ taken up by mitochondria prior to Ca^2+^ release. Ca^2+^ retention capacity values were expressed per U of COX.

### High Resolution Respirometry

Permeabilized myofiber and isolated mitochondrial respiration was assessed with a polarographic oxygen sensor (Oxygraph-2k, Oroboros, Innsbruck, Austria), calibrated as required for O_2_ concentration, environmental variables, and auto O_2_ consumption. Briefly, 3.5–6 mg (wet weight) permeabilized myofibers or 0.01 mg isolated mitochondrial protein, prepared as described above, were added to 2 ml of buffer B in the respirometer and equilibrated for baseline endogenous respiration at 37°C, with eight different animals. Myofiber respiration was measured under hyperoxygenated conditions by pre-bubbling the measurement buffer with pure O_2_ to minimize diffusion limitations at low PO_2_ in permeabilized myofibers [48]. The substrate addition protocol assessing O_2_ flux was added sequentially as follows, with each step interspersed with a period of stabilization between injections: 10 mM glutamate + 2 mM malate (GM), 2 mM adenosine di-phosphate (ADP), 10 µM succinate (SUCC), 10 µM cytochrome c, 10 µM antimycin A (AA), 5 mM ascorbate + 0.5 mM *N,N,N',N'*-tetramethyl-*p*-phenylenediamine (TMPD). Enzymatic activity measures were subsequently performed on frozen permeabilized myofibers used for respirometry experiments and freshly frozen isolated mitochondria to normalize respiration values per enzymatic unit (U) of cytochrome c oxidase (COX) activity. Respiration ratios were computed by dividing respiration values under different activation states. Respiration data is presented relative to permeabilized myofibers in the text, and the un-normalized data is available in Figure S2.

### Reactive Oxygen Species Emission

Mitochondrial H_2_O_2_ emission was measured as a surrogate for reactive oxygen species (ROS) production. Mitochondrial H_2_O_2_ and was detected by measuring the rate of appearance of resorufin, which is produced from the reaction between H_2_O_2_ and Amplex Red, with a Hitachi F-2500 fluorescence spectrophotometer at an excitation/emission wavelength of 563/587 nm, using the FL-solutions software. Samples were prepared as described above and measurements performed as described previously [45] and adapted from [49]. All measures were performed at 37°C, in duplicates for eight different animals. After the reaction was initiated, substrates were added as follows (allowing a period of stabilization between each step): GM (10+2 mM), SUCC (10 mM), ADP (10 µM), ADP (100 µM), ADP (1 mM), AA (10 µM). At the conclusion of the ROS measurements, permeabilized myofibers were placed in liquid N_2_ and stored at −80°C for enzymatic analysis. H_2_O_2_ emission is expressed as picomoles per minute per U of COX for both preparations. Data is presented relative to permeabilized myofibers in the text, and the un-normalized data is available in Figure S3.

### Biochemical assays for COX

Cytochrome c oxidase (COX) activity was used as a representative mitochondrial electron transport chain enzyme to estimate mitochondrial content in each preparation [42]. All samples were prepared in identical conditions by homogenizing isolated mitochondria (freshly frozen) and permeabilized myofibers frozen immediately after H_2_O_2_ production assay. Two samples for each of eight animals were homogenized in an extraction buffer containing 50 mM triethanolomine and 1 mM EDTA and measured in triplicates. Average COX activity values (in µmol x min^−1^ x g^−1^ of muscle) for both samples from each animal were then averaged and used to normalize functional data for each animal separately. Permeabilized myofibers were finely minced using small scissors and homogenized on ice using a small pestle rotor in 1∶20 w/v. Isolated mitochondria were diluted 1∶10 v/v, vigorously vortexed and incubated on ice for 20 minutes. COX activity was measured by detecting the decrease in absorbance at 550 nm in a 96-well plate at 30°C, using 200 µl of a reaction buffer (Potassium phosphate 100 mM, pH 7.0) containing 0.1% n-Dodecylmaltoside and 0.1 mM purified reduced cytochrome c. The molar extinction coefficients used were 13.6 L×mol^−1^×cm^−1^ for DTNB and 29.5 L×mol^−1^×cm^−1^ for reduced cytochrome c.

### Western Blotting for Electron Transport Chain Composition in Isolated Mitochondria

Under normal circumstances, mitochondrial electron transport chain (ETC) complexes are present at the inner mitochondrial membrane in a defined stoichiometry, where the relative abundance of complexes I, III and IV is expected to be well preserved [50], [51]. Frozen-thawed mitochondrial isolates and powdered whole muscle homogenates were used in Western blotting experiments to determine the relative amounts of each of the electron transport chain complexes in isolated mitochondria. Briefly, 5 µg (isolated mitochondria from 8 different animals) or 10 µg (whole muscle homogenates from 8 different animals) of protein were loaded from each isolate into precast 4–15% SDS-polyacrylamide gels (SDS-PAGE) (Bio-Rad, Hercules, USA) and ran for 1.5 h at 110 V. Proteins were then electro-transferred for 1.5 h at 400 mA onto a PVDF membrane and incubated overnight with a premixed cocktail of polyclonal antibodies directed against representative subunits of each of the electron transport chain complexes (Mitosciences MS604, 6 µg×ml^−1^; dilution 1∶1000). The antibodies recognize subunits proteins NDUFB8, CII-30, CIII-Core 2, COX-IV-1, and CV-α of Complexes I, II, III, IV, and V (ATP synthase), respectively. Equal protein loading between samples within a preparation (isolated mitochondria and whole muscle) was verified using the Ponceau red stain. Membranes were washed in 0.05% Tween-PBS buffer and incubated with horseradish peroxidase-conjugated secondary antibody (dilution 1∶5000). Signals were detected using the enhanced chemiluminescence detection system (Pierce) and chemiluminescence was digitally captured (Syngene Bio-Imager, Frederick, MD) and densitometry measured using the Bio-imager software (Syngene Tools, Frederick, MD).

### Statistical analyses

All values are presented as means ± standard error (s.e.m.). Two-tailed student's T test assuming unequal variance was used to determine P values, which were considered significant at 0.05.

## Supporting Information

Figure S1 **Raw confocal imaging data in isolated mitochondria and permeabilized myofibers.** The vast majority of isolated mitochondrial are individual spheres. Clumps or aggregates are also apparent. Both preparations were were incubated in 16.7 µM MitoTracker Red CMXROS for 20 minutes at 30°C. Protein concentration for isolated mitochondria was 2.12 mg×ml^−1^. See Videos S1 and S2 for higher resolution of morphological details of preparations of isolated mitochondria and a permeabilized myofiber.
(EPS)

Figure S2 **Absolute mitochondrial respiration values in permeabilized myofiber bundles and isolated mitochondria.**
(EPS)

Figure S3 **Absolute H_2_O_2_ production in permeabilized myofiber bundles and isolated mitochondria.** H_2_O_2_ production values are expressed per mitochondrial content (A) or per oxygen flux (JO_2_) (B).
(EPS)

Video S1 **Three-dimensional animation of a suspension of isolated mitochondria stained with Mitotracker-Red.**
(MOV)

Video S2 **Three-dimensional animation of a permeabilized myofiber stained with Mitotracker-Red.** Note that the imaged myofiber is not circular due to imaging procedure constraints.
(MOV)
